# Regional disparities in home health care utilization for older adults and their associated factors at the secondary medical area level: A Nationwide study in Japan

**DOI:** 10.1111/ggi.15011

**Published:** 2024-11-10

**Authors:** Yu Sun, Nobuo Sakata, Masao Iwagami, Satoru Yoshie, Ryota Inokuchi, Tomoko Ito, Naoaki Kuroda, Jun Hamano, Nanako Tamiya

**Affiliations:** ^1^ Department of Health Services Research Institute of Medicine, University of Tsukuba Tsukuba Japan; ^2^ Health Services Research and Development Center, University of Tsukuba Tsukuba Japan; ^3^ Department of Primary Care and Medical Education Institute of Medicine, University of Tsukuba Tsukuba Japan; ^4^ Heisei Medical Welfare Group Research Institute Tokyo Japan; ^5^ Department of Home Healthcare Setagaya Memorial Hospital Tokyo Japan; ^6^ Institute of Gerontology, University of Tokyo Tokyo Japan; ^7^ Institute for Future Initiatives, University of Tokyo Tokyo Japan; ^8^ Department of Health Policy and Management School of Medicine, Keio University Tokyo Japan; ^9^ School of Medicine, Hiroshima University Hiroshima Japan; ^10^ Department of Public Mental Health Research National Institute of Mental Health, National Center of Neurology and Psychiatry Tokyo Japan

**Keywords:** home health care services, home visit, long‐term‐care services, older people, regional disparity

## Abstract

**Aim:**

As aging populations shift health care from hospitals to communities, Japan has implemented policies to promote home health care. This study explored regional differences in home health care recipients among older adults and related factors.

**Methods:**

We used nationwide data from 2020 to describe the proportion of older adults receiving regular home visits and the medical institutions utilized across secondary medical areas: urban, middle and depopulated areas. We examined factors associated with the proportion of patients receiving regular home visits. Exposures included each secondary medical area's medical and long‐term‐care (LTC) resources, adjusted for the older adult population; proportion of single‐person households; and regional factors. We performed a multivariate negative binomial distribution analysis.

**Results:**

A total of 333 secondary medical areas were included. Urban areas had more patients receiving regular home visits, primarily from enhanced home care support clinics/hospitals (HCSCs). Fewer patients received regular home visits in depopulated areas, and conventional HCSCs were more common. Multivariate analysis revealed that the number of conventional HCSCs (coefficient, 0.17 [95% confidence intervals (CI), 0.08 to 0.26]), enhanced HCSCs (coefficient, 0.21 [95% CI, 0.14 to 0.29]) and population density (coefficient, 0.10 [95% CI, 0.02 to 0.19]) were positively associated with higher home‐visit rates. Beds in LTC welfare facilities (coefficient, −0.10 [95% CI, −0.19 to −0.01]) and beds in LTC health facilities (coefficient, −0.09 [95% CI, −0.17 to 0.00]) were negatively associated.

**Conclusions:**

Policies to promote home health care have led to high home‐visit rates in urban areas. Medical and LTC resources and regional factors influence disparities. As Japan's population ages, it is crucial to recognize these disparities and develop medical and LTC systems tailored to each region's characteristics. **Geriatr Gerontol Int 2024; 24: 1350–1361**.

## Graphical abstract

We revealed regional disparities in home‐visit rates for older adults in Japan, with urban areas having higher rates from enhanced home care clinics/hospitals, while depopulated regions rely on conventional ones or general clinics. These rates are significantly influenced by medical and long‐term care resources, as well as regional factors.

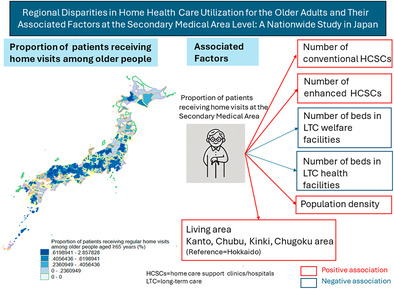

## Introduction

Population aging affects the delivery of health care and long‐term care (LTC).^1^ The demands of an aging society, complex care needs and the need to reduce medical costs have led to a global shift from hospital‐based to outpatient services.^2^ In Japan, whose rapidly aging society is the most advanced in the world,^3^ the government has promoted a shift in health care from hospitals to the community, particularly home health care. This shift has been facilitated through policies like the introduction of home care support clinics/hospitals (HCSCs) and the revision of medical fees for home health care.^4^, ^5^, ^6^ However, since the effectiveness of these measures may vary by region, it is essential to clarify the regional differences and their associated factors in home health care. Urban areas may have more abundant home health care resources and higher home health care utilization rates, whereas depopulated areas may face resource scarcity and lower utilization rates.

A systematic review reported that factors across four levels—individual, interrelationship, community and social contextual—were associated with the utilization of home‐ and community‐based services among older adults, revealing that older women, those with higher education levels, individuals in poor physical and mental health, those with caregivers and non‐White individuals had higher utilization rates than their counterparts.^7^ The results regarding place of residence are mixed, with some studies finding higher utilization rates in urban areas, while others report the opposite.^7^ Another previous study on regional differences in home‐based medical care in the United States reported significantly lower utilization rates in rural areas compared to urban areas.^8^ In Japan, several previous studies reported regional differences in home health care. For instance, a study in Hyogo Prefecture^9^ found that while there were more patients receiving regular home visits in urban areas, the number was lower in rural areas. Additionally, several nationwide studies have reported that regions with a high number of HCSCs are associated with an increased proportion of home deaths.^10^, ^11^, ^12^

However, to the best of our knowledge, no national study has focused on the proportion of regular home‐visit care. Therefore, using nationwide data, this study aimed to clarify the differences in the proportion of patients among older adults, aged ≥65 years, receiving regular home visits across secondary medical areas, as well as to identify related regional factors. These insights could help in devising strategies tailored to regional characteristics to support an aging society.

## Methods

### Home health care in Japan

All Japanese citizens have medical care coverage under a universal health insurance system. This system includes occupational insurance for employees, National Health Insurance for the self‐employed and retirees aged <75 years and a late‐stage medical care system for those aged ≥75 years.^13^ In Japan, home health care entails physicians conducting regular home visits that are covered by health insurance. In 2006, the Ministry of Health, Labour and Welfare (MHLW) introduced HCSCs.^4^, ^5^, ^6^ HCSCs offer a 24‐h home‐visiting care system at the patient's request, a standard requirement for all HCSCs. In 2012, enhanced HCSCs were introduced to provide higher‐quality home health care, focusing on emergency home visits and end‐of‐life care.^4^, ^5^, ^6^ Enhanced HCSCs must meet additional requirements: having three or more full‐time doctors, conducting at least 10 physician‐led emergency home visits in the past year and providing home‐based end‐of‐life care in at least four cases during the past year.^5^, ^6^ If a conventional HCSC meets the enhanced requirements, it can be upgraded to a higher fee category than the conventional HCSC.^4^, ^5^, ^6^ Additionally, patients who receive home visits from physicians often use nursing care and home‐help services offered by various care facilities.^14^ Despite the increasing number of HCSCs, general clinics that do not meet the HCSC requirements also provide home visits.^15^

### LTC in Japan

In 2000, Japan introduced a mandatory LTC insurance system distinct from the national medical insurance system.^16^, ^17^, ^18^ It mandates insurance premiums for all LTC services. All residents aged ≥40 years pay insurance premiums, whereas those aged ≥65 years (and those aged 40–64 years with age‐related diseases) are eligible to receive LTC services, including home‐, community‐ and facility‐based care. There are three types of facility‐based care services: LTC welfare facilities (i.e., living facilities for those who are in a stable condition), LTC health facilities (i.e., intermediate‐care facilities that aim to discharge individuals who need care and rehabilitation at home), and LTC medical facilities (i.e., medical‐based facilities for individuals who need substantial care and long‐term treatment).^17^

### Study design and data source

This was a cross‐sectional observational study. We used the National Database (NDB) Open Data to estimate the number of physician‐led home visits in secondary medical areas in Japan. The NDB is a Japanese administrative claims database provided by the MHLW that covers about 98% of the health care service data provided by health care institutions.^19^, ^20^ The MHLW publishes the “NDB Open Data Japan” online, offering several summary files by prefecture, secondary medical area, age and sex based on the NDB data.^21^ To obtain medical and LTC resources at the secondary medical area level, we used the MHLW website,^22^ where various survey data, including Vital Statistics,^23^ Survey of Medical Institutions^24^ and Survey of Institutions and Establishments for Long‐term Care,^25^ were reaggregated for each municipality. Additionally, we used original data from the Survey of Medical Institutions^24^ to obtain information on the number of hospital beds. Further, we obtained the population density of the municipalities from the 2020 population census.^26^

### Analysis unit

The analysis unit for this study was the “secondary medical area,” as defined by the Japanese government. These areas are used for planning the medical provision system and allocating resources, such as the number of medical institutions and beds.^27^ Each secondary medical area generally includes multiple municipalities. We used secondary medical areas as the unit of analysis because they are the smallest geographic units available in the NDB Open Data.

### Outcome

We defined the outcome as patients who received regular home visits, identified through the “Comprehensive Medical Management Fees at Home” from the NDB Open Data in 2020. “The Comprehensive Medical Management Fees at Home” are applicable when physicians conduct home visits for patients residing in their own homes but do not apply to patients living in residential facilities. As comprehensive management fees can be billed once per person per month, we estimated the number of individuals using regular home visits by dividing the total billings by 12, assuming 12 billings per person per year. We then calculated the proportion of older adults receiving regular home visits by dividing the estimated number of patients by the number of older adults aged ≥65 years in each secondary medical area.

### Exposure

We defined several factors to assess exposure in each secondary medical area, including medical resources, LTC facility resources, household composition and regional characteristics. Medical resources included hospital beds, general clinics, conventional HCSCs, enhanced HCSCs and home‐visiting nurses. The number of hospital beds was calculated by subtracting psychiatric beds from the total number of beds. General clinics refer to clinics that provide outpatient services and to non‐HCSC institutions, categorized as those not providing home visits and those providing home‐visit services. LTC facility resources included beds available in LTC welfare facilities, LTC health facilities and LTC medical facilities. For household composition, we used the proportion of single‐person households as a proxy for living alone. This was calculated as the number of single‐person households with older adults divided by the total number of households with older adults. Regional factors included population density and living area. Living areas were included as a variable to account for potential cultural differences in health care practices, along with differences in population density and the number of medical and LTC resources. Living areas were categorized into eight prefectural regions: Hokkaido, Tohoku, Kanto, Chubu, Kinki, Chugoku, Shikoku and Kyushu. The Kanto, Chubu and Kinki regions are considered urban owing to the presence of Japan's three major cities—Tokyo, Nagoya and Osaka—while the other regions are characterized as middle or depopulated areas.

All data were sourced from 2020. For medical and LTC facility resources, we adjusted the data by dividing by the population of older adults aged ≥65 years in each secondary medical area, expressed as “per 100 000 of the secondary medical area–level population aged ≥ 65 years” in the tables. The age of 65 years was chosen because >95% of regular home visits were conducted within this age group.^28^

### Statistical analysis

First, we demonstrated regional variations by depicting the usage rates of regular home visits among people aged ≥65 years at the secondary medical area level. Based on previous studies, we classified the secondary medical areas into three categories on the basis of population size and density: (1) urban type, characterized by a population ≥1 million or a population density ≥ 2000/km^2^; (2) middle type, characterized by a population ≥0.2 million, or a population between 0.1 and 0.2 million with a population density ≥ 200/km^2^; and (3) depopulated type, which does not fall into the above two categories.^29^ We calculated the utilization rates of regular home visits for each secondary medical area category. Additionally, we summed the estimated number of people who received regular home visits for each category and analyzed the types of medical institutions (general clinics, conventional HCSCs and enhanced HCSCs) providing services using detailed codes for “Comprehensive Medical Management Fees at Home.” We then described the characteristics of medical resources, LTC facility resources, proportion of single‐person households and regional factors for each secondary medical area category. We also described these characteristics for each living area category. Finally, we conducted a multivariate negative binomial distribution analysis with robust variance estimation to explore the factors associated with the rate of regular home visits. We used this model because overdispersion is extremely common when modeling rates and count data, and the negative binomial distribution is better suited for modeling overdispersed count data compared to the traditional Poisson regression model.^30^ All the aforementioned variables, including medical resources (hospital beds, general clinics not providing home visits, general clinics providing home visits, conventional HCSCs, enhanced HCSCs and home‐visiting nurses), LTC facility resources (beds in each of the LTC welfare facilities, LTC health facilities and LTC medical facilities), proportion of single‐person households and regional factors (population density and living area) were included in the analysis. All variables, except for the living area category, were standardized (mean = 0, standard deviation = 1) before the analyses, and standardized coefficients were interpreted.

To verify the accuracy of our estimated number of patients receiving regular home visits, we conducted a sensitivity analysis. We used data from the Statistics of Medical Care Activities in Public Health Insurance to calculate the number of times the “Comprehensive Medical Management Fees at Home” was billed in June 2020.^31^ Although these data do not offer regional specificity, it provides the monthly billing frequency of medical procedures across Japan. We compared these figures with our estimated number of home‐visit patients.

Moreover, to clarify regional disparities in all visiting medical care, we conducted an additional analysis that included both home and facility visits. We calculated the overall utilization rate of regular visiting care in each secondary medical area by incorporating the number of individuals billed for “Comprehensive Medical Management Fees at Facility,” which applies to visits made to non‐LTC facilities, like assisted living facilities with services and group homes for patients with dementia. Similar to the “Comprehensive Medical Management Fees at Home,” we estimated the number of users by dividing the total annual billing count by 12. We then combined the estimated users for both home and facility visits to calculate the total proportion of regular visiting‐care users among people aged ≥65 years in each secondary medical area. Finally, we illustrated the regional variations in these proportions and highlighted differences by secondary medical area categories, as in the main analysis.

All the analyses were performed using STATA version 17 (StataCorp, College Station, TX, USA). Statistical significance was set at *P* < 0.05.

## Results

**Figure 1:**
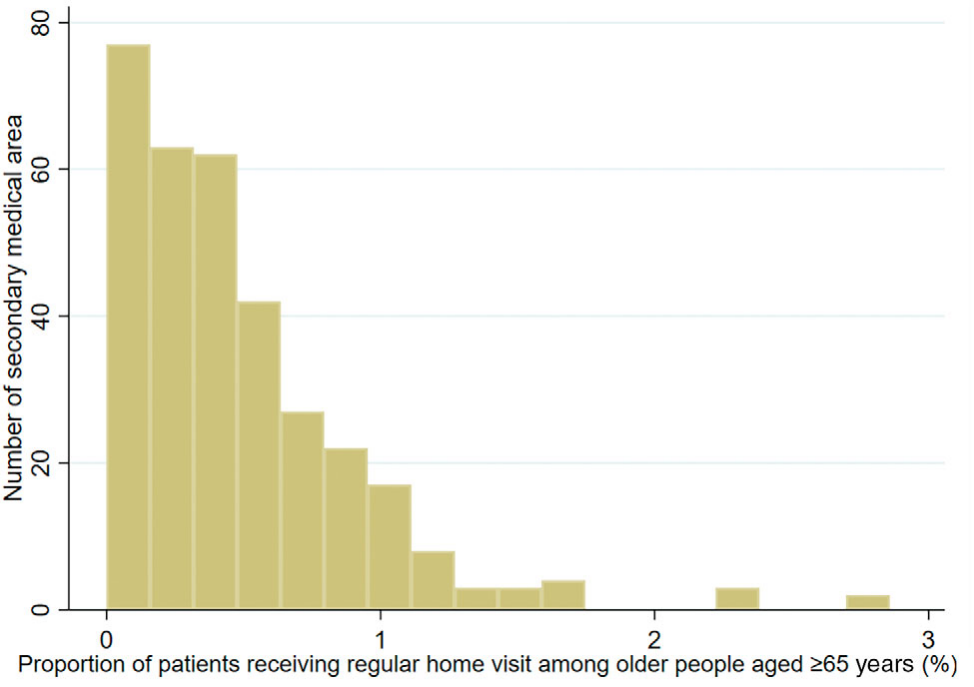
Distribution of the proportion of patients receiving regular home visits among older people aged ≥ 65 years by secondary medical area.

Excluding two secondary medical areas where data from the NDB Open Data could not be matched with the medical and LTC facilities resource database, this study encompassed 333 secondary medical areas (comprising 46 urban types, 158 middle types and 129 depopulated types). Figure 1 illustrates the proportion of patients receiving regular home visits, showcasing a right‐skewed distribution. This indicates that while the proportion of patients receiving regular home visits is low in many secondary medical areas, it is high in a few specific areas. Notably, all five secondary medical areas with a home‐visit utilization rate exceeding 2% were located in the 23 wards of Tokyo.

### Regional differences in the proportion of receiving regular home visits

**Figure 2:**
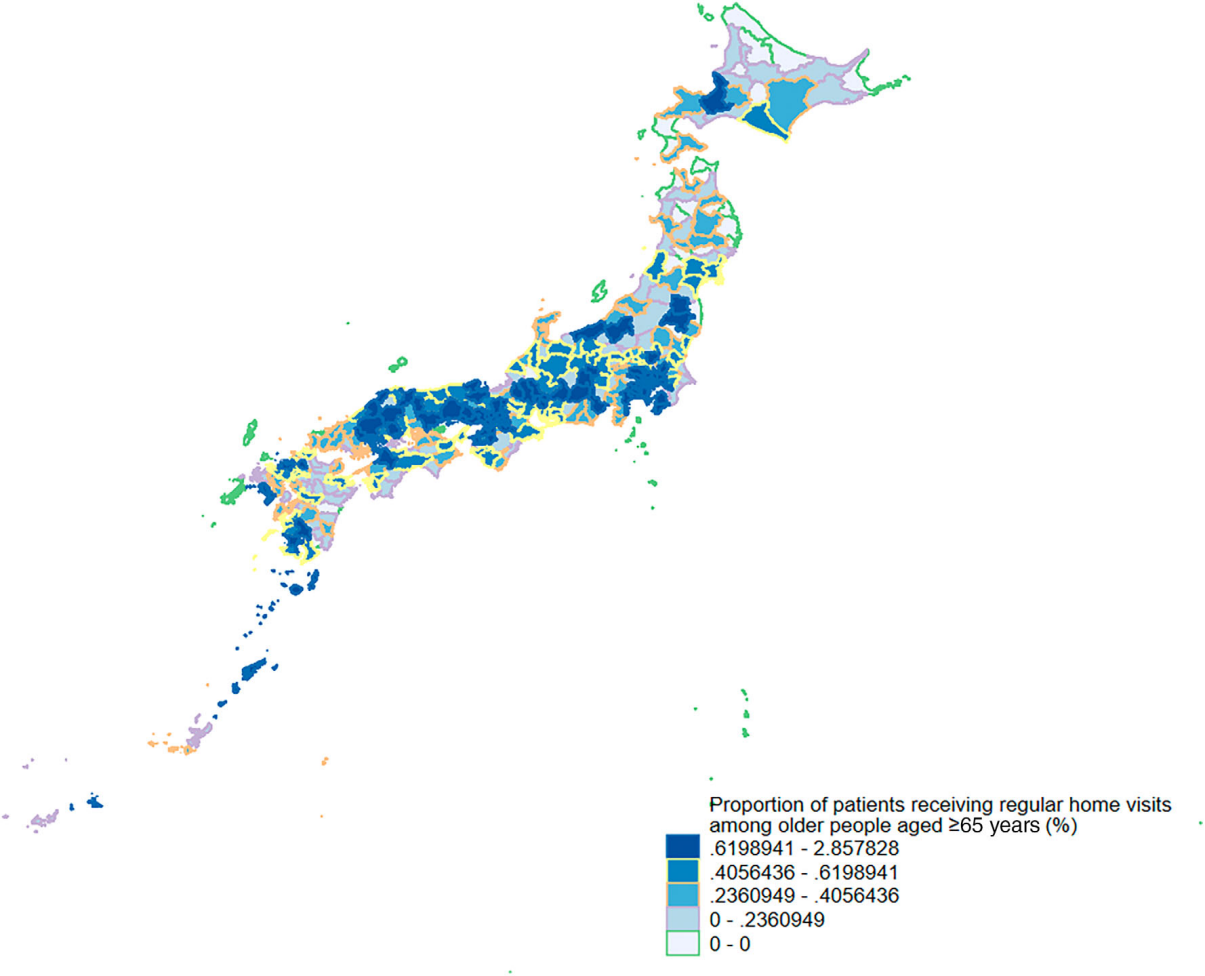
Proportion of patients receiving regular home visits among older people aged ≥ 65 years across secondary medical area nationwide.

Figure 2 displays the proportion of older adults receiving regular home visits at the secondary medical area level. It appears to be more prevalent in high‐population‐density areas such as Tokyo and Osaka. Additionally, the figure highlights regional differences, revealing lower rates in the Hokkaido and Tohoku regions and higher rates in the Chugoku region.

### Characteristics by secondary medical area category

**Figure 3:**
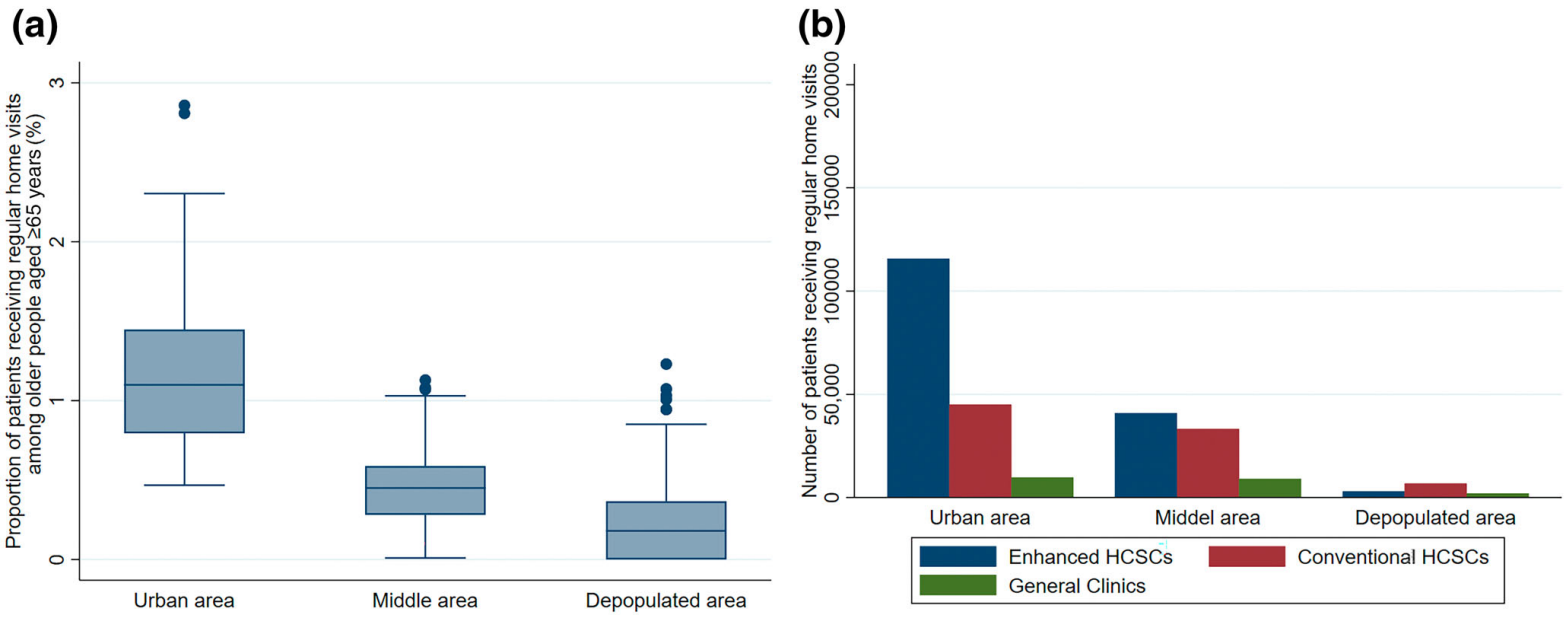
(a) Proportion of patients receiving regular home visits among older people aged ≥ 65 years by the secondary medical area categories. (b) Breakdown of the type of medical institutions providing home visits by the secondary medical area categories. The urban group includes areas with a population of 1 million or more or a population density of ≥2000 people/km^2^. The middle group included areas with a population of ≥200 000 or a population between 100 000 and 200 000, with a population density of ≥200 people/km^2^. The depopulated group includes areas that do not fall into either category. The urban type includes 46 secondary medical areas, the middle type includes 158, and the depopulated type includes 129. HCSCs, home care support clinics/hospitals; LTC, long‐term care.

Figure 3a displays the usage rate of regular home visits per older adult population when secondary medical areas were categorized into three types, while Figure 3b illustrates the breakdown of the types of medical institutions providing care. Regular home visits have the highest rates of utilization in urban areas and the lowest rates in depopulated areas. Analysis of medical institution types indicated that in urban areas, most individuals receive home visits from enhanced HCSCs, whereas in depopulated areas, most individuals rely on conventional HCSCs. Table 1 offers summary statistics for each variable across the secondary medical area categories. Conventional and enhanced HCSCs and home‐visiting nurses are more prevalent in urban areas, while general clinics offering home visits are more common in depopulated areas. Moreover, LTC welfare and health facilities tend to be more prevalent in depopulated areas.

**Table 1:** Characteristics of the secondary medical area category and overall

	Urban area	Middle area	Depopulated area	Overall
	n = 46	n = 158	n = 129	N = 333
	Median (IQR)	Median (IQR)	Median (IQR)	Median (IQR)
Proportion of patients receiving regular home visit per older adult population aged ≥65 years (%)	1.20 (0.79–1.45)	0.44 (0.28–0.59)	0.18 (0–0.37)	0.38 (0.18–0.66)
Medical resources*				
Number of hospital beds	3088 (2620–3503)	3256 (2776–3950)	2976 (2419–3584)	3170 (2608–3763)
Number of general clinics not providing home health care	203.7 (171.9–249.2)	159.4 (138.2–180.3)	133.2 (116.1–157.4)	153.6 (129.9–179.8)
Number of general clinics providing home health care	19.9 (13.0–29.4)	25.0 (16.7–34.2)	26.2 (16.4–41.6)	24.9 (16.2–34.9)
Number of conventional HCSCs	30.5 (19.9–48.0)	29.1 (18.4–43.2)	20.7 (11.8–36.1)	28.1 (16.6–41.9)
Number of enhanced HCSCs	14.9 (11.0–18.4)	7.3 (4.7–11.7)	2.1 (0–6.0)	6.4 (2.1–11.8)
Number of home‐visiting nurses	213.6 (172.0–276.4)	156.6 (116.7–198.0)	107.5 (76.7–140.3)	140.3 (102.3–192.5)
LTC resources*				
Number of beds in LTC welfare facilities	1424 (1279–1620)	1605 (1388–1883)	2053 (1780–2483)	1728 (1457–2055)
Number of beds in LTC health facilities	820.0 (765.5–1019)	1130 (987.6–1332)	1258 (1047–1419)	1153 (924.3–1347)
Number of beds in LTC medical facilities	26.1 (0–53.1)	30.5 (0–91.1)	0 (0–101.6)	26.2 (0–90.3)
Proportion of single‐person households with older adults	20.9 (18.3–23.6)	15.9 (14.4–18.3)	17.9 (15.1–21.6)	17.5 (14.9–20.7)
Population density (population per km^2^)	4822 (2738–8949)	328.7 (135.9–739.3)	67.8 (33.5–124.6)	198.7 (63.2–732.1)
Living area, *n* (%)				
Hokkaido	1 (2.2)	7 (4.4)	13 (10.1)	21 (6.3)
Tohoku	1 (2.2)	15 (9.5)	21 (16.3)	37 (11.1)
Kanto	25 (54.4)	32 (20.3)	7 (5.4)	64 (19.2)
Chubu	4 (8.7)	33 (20.9)	20 (15.5)	57 (17.1)
Kinki	12 (25.1)	19 (12.0)	14 (10.9)	45 (13.5)
Chugoku	1 (2.2)	15 (9.5)	14 (10.9)	30 (9.0)
Shikoku	0 (0)	7 (4.4)	9 (7.0)	16 (4.8)
Kyushu	2 (4.4)	30 (19.0)	31 (24.0)	63 (18.9)

* All medical and LTC resources are shown per 100 000 people aged ≥65 years.

HCSCs, home care support clinic/hospital; IQR, interquartile range; LTC, long‐term care.

### Characteristics by living area

Table A1 outlines the characteristics of medical and LTC resources by living area. The Kanto and Kinki regions, with their higher population densities, have a large number of enhanced HCSCs. In contrast, the Chugoku region, despite a lower population density, shows a relatively high utilization rate of regular home visits, characterized by numerous general clinics offering home health care and conventional HCSCs.

### Factors associated with visiting medical care

Table 2 presents the results of the multivariate negative binomial distribution analysis with robust variance estimation. The findings indicate that the number of conventional HCSCs (coefficient, 0.17 [95% confidence intervals (CI)], [0.08 to 0.26]), the number of enhanced HCSCs (coefficient, 0.21 [95% CI, 0.14 to 0.29]) and population density (coefficient, 0.10 [95% CI, 0.02 to 0.19]), show positive associations, whereas the number of beds in LTC welfare facilities (coefficient, −0.10 [95% CI, −0.19 to −0.01]) and LTC health facilities (coefficient, −0.09 [95% CI, −0.17 to 0.00]) exhibit negative associations. Furthermore, in comparison to Hokkaido, the Kanto, Chubu, Kinki and Chugoku regions exhibited a high proportion of regular home visits.

**Table 2:** Multivariable negative binomial distribution analysis with robust variance estimation for the proportion of patients receiving regular home visits

	Coefficient	95% CI	*p*
Medical resources*			
Number of hospital beds	0.05	−0.05 to 0.14	0.349
Number of general clinics not providing home health care	−0.04	−0.09 to 0.00	0.052
Number of general clinics providing home health care	0.01	−0.07 to 0.09	0.804
Number of conventional HCSCs	0.17	0.08 to 0.26	< 0.001
Number of enhanced HCSCs	0.21	0.14 to 0.29	< 0.001
Number of home‐visiting nurses	0.07	−0.02 to 0.16	0.104
LTC resources*			
Number of beds in LTC welfare facilities	−0.10	−0.19 to −0.01	0.037
Number of beds in LTC health facilities	−0.09	−0.17 to 0.00	0.043
Number of beds in LTC medical facilities	−0.02	−0.10 to 0.06	0.683
Proportion of single‐person households with older adults	0.03	−0.08 to 0.14	0.624
Population density (population/km^2^)	0.10	0.02 to 0.19	0.017
Living area			
Hokkaido	Reference	Reference	Reference
Tohoku	0.41	−0.15 to 0.97	0.150
Kanto	1.01	0.50 to 1.51	< 0.001
Chubu	0.79	0.28 to 1.31	0.002
Kinki	0.76	0.24 to 1.29	0.004
Chugoku	0.73	0.22 to 1.24	0.005
Shikoku	0.41	−0.11 to 0.93	0.124
Kyushu	0.29	−0.23 to 0.80	0.277

* All medical and LTC resources were divided by100 000 people aged ≥65 years.

All variables, except living area, were standardized before analysis, and the standardized coefficients were interpreted.

HCSCs, home care support clinics/hospitals; LTC, long‐term care.

### Sensitivity analysis

In the sensitivity analysis, we compared our estimated patient numbers with data from the Statistics of Medical Care Activities in Public Health Insurance. In June 2020, 272 205 individuals were billed for Comprehensive Medical Management Fees at Home across Japan. This closely matches our estimate of 288 816 patients, which was calculated by dividing the total annual count from the NDB Open Data by 12.

### Additional analysis

Appendix A2 shows the proportion of regular visiting medical care among older people by secondary medical areas, including facility residents. Compared to Figure 1, which focuses only on home residents, the Kyushu region saw an increase, indicating more facility visits in this area. The distribution in other regions remained consistent. Additionally, when secondary medical areas were classified into three types, the proportions of regular visit care and breakdown of providing institutions were similar to the main analysis, with a significant number of people in urban areas receiving care from enhanced HCSCs (Appendices A3 and A4).

## Discussion

To our knowledge, this study represents the first national‐level investigation into regional disparities in the utilization rates of regular home visits across secondary medical areas, along with an exploration of associated factors. It unveils distinct regional gaps in the utilization of regular home visits and the distribution of medical institution types providing care, contingent upon the population density and population size of the secondary medical area. Multivariate analysis revealed a positive association between HCSCs and population density with high rates of regular home visits, while a negative correlation was observed with LTC facilities.

Our finding of higher rates of regular home visits in areas with greater population density aligns with those of previous studies in foreign countries.^7^, ^8^ A previous study noted a moderate correlation between population density and the number of home health care providers, especially for enhanced HCSCs,^10^ suggesting that urban locales possess greater resources for providing home health care, thus yielding higher utilization rates. Another possibility is that differences in the attributes of family caregivers between rural and urban areas, as well as cultural differences regarding the choice of institutional care, may be relevant.^32^, ^33^ We also identified regional differences, with lower rates in the Hokkaido and Tohoku regions, which are reported to face challenges in securing hospital beds for sudden patient deterioration and experience higher burdens on home health care.^34^ Contrastingly, the Chugoku region displayed higher rates, characterized by a large number of general clinics providing home health care and conventional HCSCs. This aligns with previous surveys indicating that many clinics in the Chugoku region conduct home visits themselves when outpatient care patients require them.^34^ These findings suggest that cultural differences in health care practices may influence home‐visit rates across regions.

Upon categorizing by secondary medical area, urban areas had a higher number of patients receiving regular home visits, predominantly from enhanced HCSCs. Conversely, in depopulated areas, fewer patients received regular home visits, with conventional HCSCs being more prevalent. Additionally, while depopulated areas have fewer home health care resources like HCSCs and home‐visiting nurses, they tend to have more LTC facilities. This aligns with previous findings indicating that urban areas have more in‐home service users, while rural areas favor community‐ and facility‐based services.^35^ This is because, besides the choices made by patients and their families, areas with low population densities are at a disadvantage for home visits due to widely spaced residences,^36^ leading to difficulties in recruiting physicians in enhanced HCSCs, which require at least three full‐time physicians. In addition, the limited availability of other home‐visiting services in depopulated areas may also contribute to the lower utilization of home health care. Instead, conventional HCSCs or general clinics, typically run by single practitioners,^36^ support home health care in depopulated areas.

Our finding of a negative association between the number of LTC welfare facilities and LTC health facilities and regular home visits can be attributed to two main reasons: first, having an adequate number of LTC facilities may reduce the likelihood of patients receiving home‐visit care; and second, the inability to sustain care at home owing to a lack of home care resources necessitates the establishment of more LTC facilities.

Our study shows that urban areas have well‐equipped home care facilities and high rates of home visits, while depopulated areas, with fewer home visits, may have more older people living in LTC facilities. The additional analysis, which included facility visits, confirmed that aside from the high frequency of facility visits in the Kyushu region, there were minimal changes. This suggests that while some areas may substitute home visits with facility visits, in many regions, the pattern of facility visits mirrors that of home visits. This implies that even in urban areas, a lack of family caregiving may lead to a greater number of older adults living in facilities, resulting in higher rates of regular visits to these residents.

With the nationwide increase in the older adult population and an anticipated shortage of family caregivers due to declining birth rates and the prevalence of nuclear family structures, the necessity of ensuring adequate facilities for the older adults in depopulated areas, along with the caregiving staff who operate within them, becomes increasingly paramount. Moreover, to sustain support for home care in alignment with patient and family preferences, even in depopulated areas, new policies may be necessary. These could include increasing home health care resources by implementing additional fees for enhanced HCSCs based on population density, enhancing telemedicine systems, advocating delegation to other health care professionals and promoting an integrated care program including both home visits and overnight stays. Furthermore, considering that the Chugoku region has a notably high rate of home health care utilization despite the challenges posed by low population density, there may be systems in place—possibly led by regional medical associations—that facilitate easier coordination among clinics to provide home visits, even without enhanced HCSCs. Further analysis is required to understand how these clinics deliver effective home health care services.

This study has some limitations. First, although there may be variations in the disease and care needs of patients receiving home visits depending on the region, we were unable to consider patients' backgrounds. Additionally, patient backgrounds may vary by medical institution type. Therefore, while this study assumed that patients received home visits for 1 year, those who were unstable may receive visits only for a shorter period. In regions where many patients have a short duration of home health care, the total annual utilization rate of home health care is expected to increase. However, a previous study finds little variation in the number of patients who died within 6 months of starting to receive home visits, regardless of regional size, suggesting that regional differences in the duration of care are minimal and likely have a limited impact on the results.^36^ Moreover, the sensitivity analysis reveals that the home health care utilization rate in June 2020 closely matches that of our study's estimate. This indicates that the duration of care is not a significant factor if we consider the June 2020 rate as the outcome. Second, we were unable to obtain information on facilities other than LTC facilities (e.g., assisted living facilities). Third, although this study analyzed data at the level of secondary medical areas, which are the smallest geographic units available in the NDB Open Data, these areas include geographically diverse regions with varying medical resources. Future studies may need to analyze smaller units, such as municipalities, to develop more tailored strategies that consider specific regional characteristics.

In conclusion, leveraging comprehensive national empirical data, we observed regional disparities in the rates of regular home visits. Regular home visits were more prevalent in areas with abundant enhanced HCSC resources and high population densities, and were less common in regions with numerous LTC facilities. Furthermore, the proportions were demonstrated to vary according to the living area. As the aging population continues to grow across Japan, the significance of recognizing these disparities and developing medical and LTC systems tailored to the characteristics of each region has been highlighted.

## Disclosure statement

The authors declare no competing interests.

## Author contributions

Study concept and design: YS, NS, RI, MI, JH. Statistical analysis and interpretation of data: YS and NS. Preparation of the manuscript: All authors contributed to drafting and critical revision of the manuscript. All authors approved the final manuscript.

## Ethics statement

No ethical approval was obtained, as only publicly available data were used.

## Figures and Tables

**Appendix A2:**
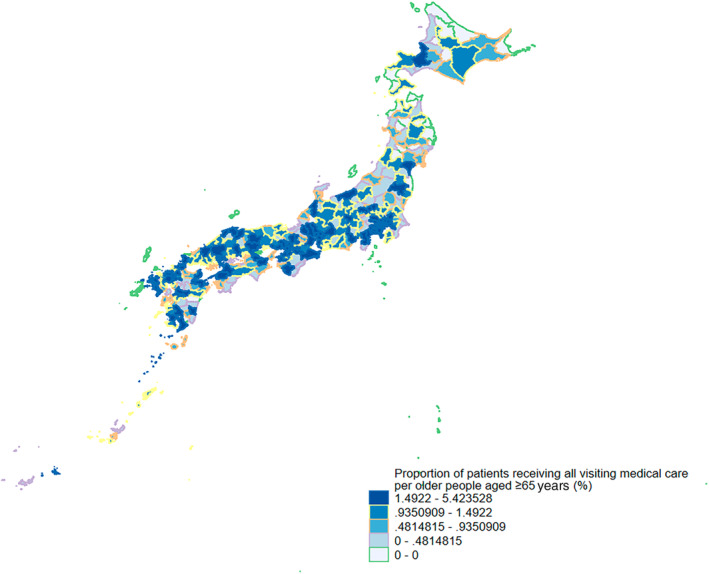
Proportion of patients receiving all regular visiting medical care (including both home and facilities) per older people across secondary medical areas nationwide.

**Appendix A3:**
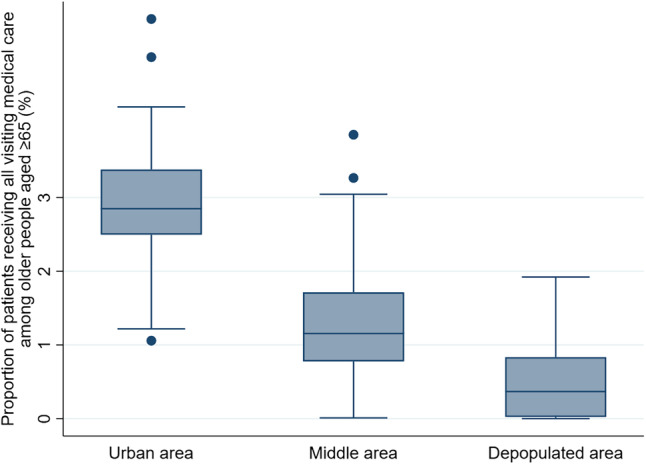
Proportion of patients receiving all regular visiting care (including both home and facilities) per older people by the secondary medical area categories. The urban group includes areas with a population of 1 million or more or a population density of 2000 people per km^2^ or more. The middle group included areas with a population of 200 000 or more or a population between 100 000 and 200 000, with a population density of 200 people/km^2^ or more. The depopulated group includes areas that do not fall into either category. The Urban type includes 46 secondary medical areas, the Middle type includes 158, and the Depopulated type includes 129.

**Appendix A4:**
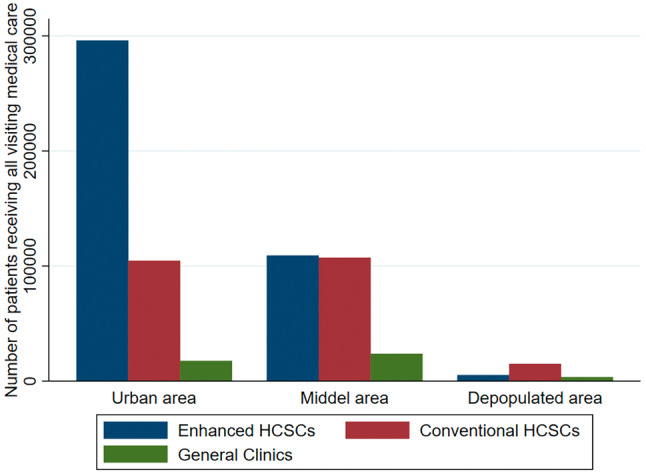
Breakdown of the type of medical institutions providing all visiting medical care (including both home and facilities) by the secondary medical area categories. HCSCs: Home care support clinics and hospitals; The urban group includes areas with a population of 1 million or more or a population density of 2000 people per km^2^ or more. The middle group included areas with a population of 200 000 or more or a population between 100 000 and 200 000, with a population density of 200 people/km^2^ or more. The depopulated group includes areas that do not fall into either category. The Urban type includes 46 secondary medical areas, the Middle type includes 158, and the Depopulated type includes 129.

## Data Availability

All data used in this study are publicly available. The data can be accessed from the following sources: NDB Open Data https://www.mhlw.go.jp/stf/seisakunitsuite/bunya/0000177182.html
Regional data collection for home health care https://view.officeapps.live.com/op/view.aspx?src=https%3A%2F%2Fwww.mhlw.go.jp%2Fcontent%2F10800000%2F001316849.xlsx&wdOrigin=BROWSELINK https://view.officeapps.live.com/op/view.aspx?src=https%3A%2F%2Fwww.mhlw.go.jp%2Fcontent%2F10800000%2F001094335.xlsx&wdOrigin=BROWSELINK
Population census in 2020 https://www.e‐stat.go.jp/stat‐search/database?page=1&query=%E4%BA%BA%E5%8F%A3%E5%AF%86%E5%BA%A6&layout=dataset&toukei=00200521&tstat=000001136464&collect_area=200&metadata=1&data=1
Survey of medical institutions in 2020 https://www.e‐stat.go.jp/stat‐search/database?page=1&layout=datalist&toukei=00450021&tstat=000001030908&cycle=7&tclass1=000001165107&tclass2=000001165167&tclass3=000001165170&collect_area=200&tclass4val=0&metadata=1&data=1

## Appendix A

**Table A1:** Characteristics of each living area

	Hokkaido	Tohoku	Kanto	Chubu	Kinki	Chugoku	Shikoku	Kyushu
	N = 21	N = 37	N = 64	N = 57	N = 45	N = 30	N = 16	N = 63
	Median (IQR)	Median (IQR)	Median (IQR)	Median (IQR)	Median (IQR)	Median (IQR)	Median (IQR)	Median (IQR)
Proportion of patients receiving regular home visit per older adult population (%)	0.09 (0–0.24)	0.16 (0–0.35)	0.62 (0.28–1.08)	0.45 (0.26–0.56)	0.64 (0.45–0.94)	0.46 (0.35–0.65)	0.33 (0.08–0.48)	0.28 (0.11–0.48)
Proportion of patients receiving all regular visiting care (including both home and facilities) per older adult population (%)	0.23 (0–0.61)	0.31 (0–0.94)	1.46 (0.62–2.92)	1.04 (0.42–1.48)	1.23 (0.69–2.46)	1.22 (0.95–1.71)	0.52 (0.32–1.45)	0.96 (0.44–1.70)
Medical resources*								
Number of hospital beds	4013 (3206–4482)	2817 (2225–3232)	2745 (2440–3285)	2767 (2272–3249)	3254 (2898–3530)	3660 (3248–3976)	3570 (3001–3803)	3810 (3087–4687)
Number of general clinics not providing home healthcare	129.9 (118.3–143.8)	150.0 (131.4–163.4)	177.2 (145.9–206.8)	151.8 (124.0–172.8)	161.9 (141.9–186.0)	146.9 (131.0–172.4)	143.9 (127.3–166.5)	150.6 (119.7–185.9)
Number of general clinics providing home healthcare	13.2 (9.5–18.2)	17.0 (13.0–28.2)	16.1 (11.4–20.8)	27.4 (21.5–40.8)	38.9 (29.2–46.2)	40.0 (28.9–49.3)	25.7 (18.3–31.3)	23.9 (18.3–31.7)
Number of conventional HCSCs	9.3 (4.8–15.5)	16.8 (10.6–19.9)	20.5 (14.4–29.4)	26.3 (17.4–31.9)	40.4 (31.1–48.3)	40.8 (27.1–61.5)	26.3 (17.5–46.6)	39.0 (28.5–52.1)
Number of enhanced HCSCs	3.1 (0–5.6)	1.8 (0–6.9)	8.9 (5.0–14.7)	5.8 (3.0–11.5)	11.00.96 (5.5–16.1)	5.8 (1.2–14.5)	7.7 (3.3–11.4)	5.3 (0–11.7)
Number of home‐visiting nurses	101.6 (70.0–134.0)	95.2 (69.5–116.7)	140.7 (111.0–185.6)	138.6 (105.2–174.8)	199.3 (169.4–261.8)	144.0 (111.3–199.7)	114.5 (93.1–146.0)	167.7 (115.8–210.0)
LTC resources*								
Number of beds in LTC welfare facilities	1986 (1603–2548)	1832 (1680–2046)	1676 (1455–1948)	1812 (1479–2050)	1706 (1360–2157)	1729 (1465–2368)	1707 (1470–2002)	1670 (1438–1933)
Number of beds in LTC health facilities	971 (870–1211)	1355 (1215–1540)	1015 (786–1165)	1180 (1065–1355)	1004 (789–1215)	1176 (936–1365)	1144 (954–1552)	1266 (1014–1425)
Number of beds in LTC medical facilities	34.1 (0–90.1)	9.91 (0–42.0)	3.92 (0–59.7)	22.1 (0–60.0)	12.3 (0–52.7)	25.2 (0–94.0)	101 (18.6–164)	78.9 (0–137)
Proportion of single‐person households with older adults	22.1 (20.5–22.6)	15.5 (13.1–17.0)	17.3 (15.4–19.5)	14.6 (13.6–15.9)	17.7 (15.6–20.9)	18.2 (16.0–20.5)	20.7 (18.2–21.5)	19.3 (16.2–22.4)
Population density (population per km^2^)	11.9 (8.3–20.3)	75.8 (34.3–154)	1197 (317–6440)	256 (87.2–645)	466 (136–1840)	86.8 (51.6–246)	185 (38.8–395)	177 (74.0–501)

* All medical and LTC resources are shown per 100 000 people aged 65 years and older.

Abbreviations: IQR, interquartile range; HCSCs, home care support clinic and hospital; LTC, long‐term care.
